# Effectiveness of interventions using self-monitoring to reduce sedentary behavior in adults: a systematic review and meta-analysis

**DOI:** 10.1186/s12966-019-0824-3

**Published:** 2019-08-13

**Authors:** Sofie Compernolle, Ann DeSmet, Louise Poppe, Geert Crombez, Ilse De Bourdeaudhuij, Greet Cardon, Hidde P. van der Ploeg, Delfien Van Dyck

**Affiliations:** 1 Department of Movement and Sport Sciences Faculty of Medicine and Health Sciences, Ghent University, Watersportlaan 2, B-9000 Ghent, Belgium; 2 Research Foundation Flanders (FWO), Brussels, Belgium; 3 Department of Experimental Clinical and Health Psychology, Ghent University, Ghent, Belgium; 4 Department of Public and Occupational Health Amsterdam Public Health research institute Amsterdam UMC, Vrije Universiteit Amsterdam, Amsterdam, the Netherlands

**Keywords:** Sitting time, Sedentary time, Electronic devices, Efficacy, Program

## Abstract

**Background:**

Sedentary behavior occurs largely subconsciously, and thus specific behavior change techniques are needed to increase conscious awareness of sedentary behavior. Chief amongst these behavior change techniques is self-monitoring of sedentary behavior. The aim of this systematic review and meta-analysis was to evaluate the short-term effectiveness of existing interventions using self-monitoring to reduce sedentary behavior in adults.

**Methods:**

Four electronic databases (PubMed, Embase, Web of Science, and The Cochrane Library) and grey literature (Google Scholar and the International Clinical Trials Registry Platform) were searched to identify appropriate intervention studies. Only (cluster-)randomized controlled trials that 1) assessed the short-term effectiveness of an intervention aimed at the reduction of sedentary behavior, 2) used self-monitoring as a behavior change technique, and 3) were conducted in a sample of adults with an average age ≥ 18 years, were eligible for inclusion. Relevant data were extracted, and Hedge’s g was used as the measure of effect sizes. Random effects models were performed to conduct the meta-analysis.

**Results:**

Nineteen intervention studies with a total of 2800 participants met the inclusion criteria. Results of the meta-analyses showed that interventions using self-monitoring significantly reduced total sedentary time (Hedges g = 0,32; 95% CI = 0,14 − 0,50; *p* = 0,001) and occupational sedentary time (Hedge’s g = 0,56; 95% CI = 0,07 − 0,90; *p* = 0,02) on the short term. Subgroup analyses showed that significant intervention effects were only found if objective self-monitoring tools were used (g = 0,40; 95% CI = 0,19 − 0,60; *p* < 0,001), and if the intervention only targeted sedentary behavior (g = 0,45; 95% CI = 0,15-0,75; *p* = 0,004). No significant intervention effects were found on the number of breaks in sedentary behavior.

**Conclusions:**

Despite the small sample sizes, and the large heterogeneity, results of the current meta-analysis suggested that interventions using self-monitoring as a behavior change technique have the potential to reduce sedentary behavior in adults. If future – preferably large-scale studies – can prove that the reductions in sedentary behavior are attributable to self-monitoring and can confirm the sustainability of this behavior change, multi-level interventions including self-monitoring may impact public health by reducing sedentary behavior.

**Electronic supplementary material:**

The online version of this article (10.1186/s12966-019-0824-3) contains supplementary material, which is available to authorized users.

## Background

Adults’ sedentary behavior levels are high in developed countries [1, 2]. A study pooling accelerometer data from adults (aged between 20 and 75 years) of four different European countries revealed an average sedentary time of 8.83 h/day [3]. Another study, in which results of accelerometer-measured sedentary time in older adults (aged above 60 years) were summarized, showed a daily mean of 9.40 h/day [4]. Reducing these high prevalence rates is a public health priority, as excessive sedentary behavior is associated with a plethora of negative health outcomes, ranging from non-communicable diseases (e.g. cardiovascular disease and type II diabetes) and poor mental health, to all-cause mortality [5–8]. Although no international consensus regarding specific guidelines for adults’ sedentary behavior has been reached, a meta-analysis conducted by Chau and colleagues suggested that adults’ sedentary time should be limited to 7 to 8 h/day [9]. Apart from general advise to reduce sedentary behavior, several national public health guidelines have also recommended to break up sedentary time every 30 min [10–12].

Unfortunately, evidence on effective intervention strategies to target adults’ sedentary behavior is still limited [5, 10, 13]. Existing sedentary behavior interventions have mainly focused on reducing occupational sedentary behavior, whereas there is a lot to be gained in other domains of sedentary behavior. Leisure time for example is proportionally the most sedentary domain [14]. Moreover, they have been largely informed by social-cognitive models of behavioral change (e.g. Theory of Planned Behavior) [15, 16]. Most of these models are based on an expectancy-value framework in which behavior is determined by expected outcomes and the value that is placed on them [17]. As such, these models do not adequately capture processes underlying unintentional and habit-like behavior. Given that a large part of sedentary behavior is habitual (i.e. it involves little reasoning and is performed without conscious decision making) [18], specific behavior change techniques (BCTs) are needed to better control sedentary behavior. One powerful strategy to disrupt habits is to change the circumstances, so that habit cueing does not occur anymore [19], or to alter the external cues that lead to habit execution [20]. However, these strategies have practical difficulties, since manipulating or avoiding cues is often impossible, or not always seen as ethical [21, 22]. Therefore, another way to disrupt undesired habits is preferred, namely by bringing habitual behavior and its context into conscious awareness. This might be achieved by means of self-monitoring [21].

Self-monitoring – defined by Michie and colleagues as keeping a record of a specified behavior as a method for changing behavior [23] – has been identified in the review of Gardner et al. as a promising behavior change technique to reduce sedentary behavior in adults [15]. Within the review of Gardner, the aim was to consider how sedentary behavior in adults might best be reduced, by describing the behavior change strategies used in sedentary behavior reduction intervention evaluations. However, it should be noted that the eligibility of interventions in the latter review was dependent on outcomes, such that any behavior change intervention was eligible where primary quantitative data were available in at least one indicator of sedentary behavior. Interventions that did not explicitly target sedentary behavior were thus included if sedentary behavior data were available. Given previous mentioned eligibility criteria, and the fact that research on sedentary behavior was still in its infancy at the time of the review, the majority of the studies included in review of Gardner aimed to increase physical activity rather than to reduce sedentary behavior [15]. As such, most of the included intervention studies used a pedometer as a self-monitoring tool [15].

More recently, bodily worn electronic devices, that allow to self-monitor sedentary behaviors, have emerged as a result of technological advances. These electronic devices have reduced the burden (i.e. time and task demand) of traditional paper-based methods and have increased assessment accuracy, which might have resulted in improved adherence, and in turn, greater achievement of sedentary behavior goals [24].

Despite the large potential for behavior change and the rise of bodily worn electronic devices to self-monitor sedentary behavior, it remains unclear how effective this behavior change technique is to reduce sedentary behavior in adults. Therefore, this study aimed to systematically review and evaluate the existing evidence regarding the effectiveness of interventions using self-monitoring to reduce sedentary behavior in adults by means of a meta-analysis. Additionally, the current meta-analysis aimed to identify factors moderating the observed effectiveness. Firstly, the dose-response relationship will be explored by testing the moderating effect of the intervention duration. Based on previous physical activity research, it can be hypothesized that longer interventions might yield better results [25]. Secondly, the moderating effect of the specific self-monitoring tool will be assessed. Given that paper-based diaries or online recording forms of sedentary behavior are time-consuming, and often subject to error and recall bias [26], we expect higher effect sizes in intervention studies using objective self-monitoring tools. Moreover, better effects are expected from tools specifically developed to monitor sedentary behavior, compared to tools in which the main aim is to track physical activity. Thirdly, it will be examined if age acts as a moderator in the observed effectiveness. Considering the higher sedentary time [27], it might be expected that older adults have more potential to reduce their sedentary time. However, the stronger habits developed for sedentary behavior [16], and the experienced difficulties both while working with electronic devices [28], and by standing – due to for example pain, fatigue, and functional limitations [29, 30] – might be important barriers for older adults to reduce their sedentary time by means of self-monitoring. Fourthly, the moderating effect of health status will be tested, as it might be that participants with overweight/obesity or other clinical conditions might be more motivated to reduce their sitting time or find it harder. Fifthly, the intervention content will be included as a moderator in order to examine if self-monitoring in itself is enough to achieve behavior change or if it should be combined with other behavior change techniques. Finally, the focus of the intervention will be included as a moderator. Previous research has suggested that interventions targeting only sedentary behavior have a larger impact compared to interventions targeting both physical activity and sedentary behavior [31].

## Methods

This review was performed in accordance with the PRISMA (Preferred Reporting Items for Systematic Reviews and Meta-analyses) guidelines (see Additional file 1). The protocol of this review (see Additional file 2) was registered with PROSPERO, which is an internationally database of prospectively registered systematic reviews in health and social care (registration ID: CRD42018112735).

### Data sources and search strategy

A systematic literature search of four electronic databases (PubMed, Embase, Web of Science, and The Cochrane Library) was performed in October 2018 – and updated at the end of May 2019 – to detect intervention studies meeting the inclusion criteria. Additionally, forward and backward reference checking of the included papers was applied, and grey literature was searched – as recommended in the current Cochrane Collaboration guidelines – using Google Scholar, and the International Clinical Trials Registry Platform. If an intervention trial was detected using the grey literature search, authors were contacted to request unpublished data. The search strategy was developed using the PICO (population, intervention, comparison, outcome) acronym. The population of interest was adults, the intervention included self-monitoring, the comparison group received no intervention or an intervention without self-monitoring, and (one of) the study primary outcome(s) was sedentary behavior. The search was limited to articles published in English between the beginning of 2000 and the end of May 2019. This start date was chosen since most older studies used the construct sedentary behavior as a synonym for physical inactivity. Details on the search strategy – which was adapted to the specific features of each database – are presented in Table 1.

**Table 1:** Search strategy Pubmed

Building blocks	Search terms
	intervention OR trial OR effectiveness OR efficacy
AND	“sedentary behavior” OR “sedentary behaviour” OR “sedentary behaviors” OR “sedentary behaviours” OR “sedentary time” OR “sedentary lifestyle” OR “sitting time” OR “TV time” OR “TV viewing” OR “watching TV” OR “computer time” OR “computer use” OR “screen time” OR “sedentary activity” OR “sedentary activities” OR driving OR “passive transport” OR “car use” OR “motor transport” OR gaming
AND	adult OR individuals OR adults OR elderly OR aged OR “older people” OR seniors OR senior OR workers OR employees OR men OR women OR patients OR survivors

After running the search strategy, duplicates were removed. Subsequently, the studies were screened by title, and abstract by the first author (SC). After this first selection, full texts were independently screened by two reviewers (SC and DVD) to determine their eligibility based on the inclusion criteria. When doubt regarding the inclusion of a study persisted, a third reviewer (ADS) was consulted.

### Study selection

Studies were eligible for inclusion if they were conducted in adults with an average sample age of 18 years and above, and if they were assessing the short-term effectiveness of sedentary behavior interventions, included self-monitoring as a behavior change technique, and used a controlled intervention trial design (i.e. [cluster-]randomized controlled trial or non-randomized controlled trial). Short-term effectiveness was defined as effectiveness reported immediately post intervention. Interventions were considered to use self-monitoring as a behavior change technique, if the participants were asked to keep a record of their sedentary behavior or physical activity as a method for changing behavior [23]. Self-monitoring had to be an explicitly stated behavior change technique, as opposed to occurring as part of completing measures for research purposes. This could for example take the form of a diary, completing a questionnaire about their behavior, in terms of type, frequency, duration and/or intensity, and/or the use of an electronic device. Interventions were included in the current review if they aimed at either 1) the reduction of total sedentary behavior, 2) the reduction of domain-specific sedentary behavior (e.g. occupational sedentary behavior, leisure time sedentary behavior), or 3) the increase in the number of sedentary behavior interruptions. Interventions in which the sole aim was to increase physical activity were excluded as previous research has indicated that these interventions revealed no, or only small effects on sedentary behavior [13].

### Data extraction

Data were extracted from the published articles when available and authors were contacted to request missing data. Data extraction was done by SC and DVD independently using a standardized form and included: source characteristics (i.e. author, year of publication, and country of publication); study design; sample characteristics (i.e. sample size, age, gender, healthy vs clinical); intervention characteristics (i.e. duration, focus, self-monitoring, other behavior change techniques,); control characteristics (i.e. no intervention or other behavior change techniques); sedentary behavior measure(s) (i.e. objective vs self-reported, total sedentary behavior vs domain-specific sedentary behavior and measurement instrument) and sedentary behavior outcome data (i.e. the means and standard deviations of each group for both pre and post assessment, or mean changes and SD differences, or F values for group differences between changes). Follow-up measurements were not extracted, as the majority of the included studies did not report/analyze long-term effectiveness. Consensus was used to resolve disagreement regarding the data extraction. If consensus could not be reached, inconsistencies were discussed with a third reviewer (ADS).

### Quality assessment

Methodological study quality was assessed using the Effective Public Health Practice Project (EPHPP) Quality Assessment Tool for quantitative studies (https://merst.ca/ephpp/). Studies were independently reviewed by two researchers (SC and LP) and disagreements were resolved through discussion. Each of the following aspects were rated as weak, moderate or strong, using the EPHPP: selection bias, study design, confounders, blinding, data collection methods, withdrawals and drop-outs, intervention integrity and analyses. Because blinding of participants was not feasible in the context of self-monitoring based interventions aimed at the reduction of sedentary behavior, the overall risk of bias was calculated for each study without taking into account the blinding score. The impact of the study quality on overall effects was assessed by sensitivity analyses (see below).

### Data analysis

#### Meta-analyses

Meta-analyses were conducted using Comprehensive Meta-analyses software version 3.3.070 (Biostat Inc., Englewood, NJ, USA). For each study an effect size was calculated with Hedges’ formula correcting for small samples [32]. By calculating Hedge’s g, all effect sizes were transformed to a common metric, which enabled us to include different outcome measures in the same analysis [33]. Subsequently, the unadjusted difference in means was calculated using the absolute time spent sedentary (min/day) as an effect size to gain insight into the difference in sedentary time between the intervention and the control groups. Random effects models were used for the meta-analyses. The random effects models estimated the mean of a distribution of effects [33]. Findings of the meta-analysis were presented using forest plots.

### Test of heterogeneity and moderation analyses

The existence of heterogeneity was assessed using the Cochrane’s Q test, and the I^2^ statistics. A Q-value with a significance of *p* ≤ 0.05 was considered significant heterogeneity, while for the I^2^, a cut-off point of 50% was considered indicative of high heterogeneity [34]. If high heterogeneity was present, moderator analyses were conducted to test whether the heterogeneity could be explained by differences in 1) the intervention duration (≤ 12 weeks vs >  12 weeks), 2) the main purpose of the self-monitoring tool (sedentary behavior tool vs physical activity tool), 3) the way of self-monitoring (self-reported vs objective monitoring), 4) the age group of the participants (mean age ≤ 60 years vs mean age > 60 years) 5) the health status of the participants (healthy vs overweight/obese or other clinical condition), 6) the intervention content (only self-monitoring and general information on the link between behavior and health outcome vs combined with other behavioral change techniques) and 7) the focus of the intervention (only sedentary behavior or multiple behaviors). The median was used to calculate the cut-off point for intervention duration and mean age. The main purpose of the self-monitoring tool was determined using websites from manufacturers and the scoping review of Sander et al. [35] (see Additional file 2).

### Sensitivity analyses and publication bias check

Sensitivity analyses were carried out to check the robustness of the statistical analysis. Concretely, main analyses were repeated without the study of Wyke et al. [36], as this study might have affected the results due to the large sample size and hence large contribution to the meta-analysis. Main analyses were also repeated without low quality studies, without cluster-randomized controlled trials, and without studies using subjective measurement instruments. The presence of publication bias was assessed using a funnel plot and Egger’s regression test.

## Results

### Study characteristics

**Fig. 1:**
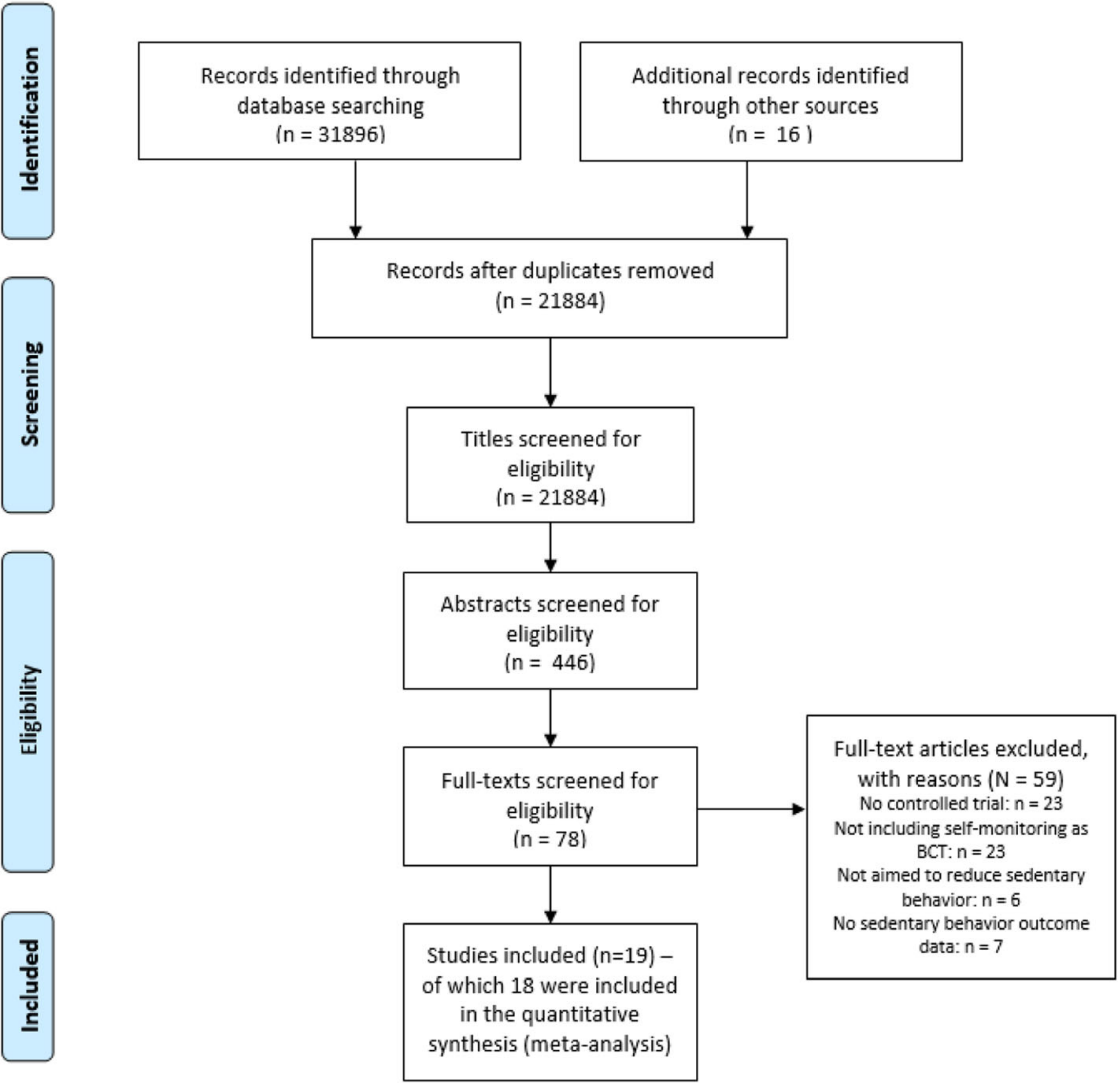
PRISMA flowchart

Figure 1 displays the number of studies identified, screened and excluded at each stage of the review process using the PRISMA flowchart. As a result, 19 articles met the inclusion criteria [36–52]. Of these, one study [53] was not included in the quantitative syntheses, as information was only provided on the longest bout of sedentary behavior.

Characteristics of the included studies are presented in Table 2. The majority of the included studies used a pretest posttest control group design (11/19) [36, 37, 39–41, 44, 46, 48, 50, 52, 54]. Five studies used a non-equivalent pretest posttest control group design [42, 45, 47, 49, 55], and three studies used a multigroup pretest posttest design [43, 51, 53]. Of the included studies, six studies were conducted in the USA [41, 46, 48, 50, 51, 55], four in the UK [40, 45, 49, 52], three in Belgium [43, 44, 54], two in Australia [37, 42] and one each in Canada [39], Japan [53] and Taiwan [47]. One study was conducted in multiple countries [36]. Only five studies were conducted in older adults (i.e. mean age above 60 years) [37, 39, 44, 48, 52], whereas 14 studies were conducted in adults (i.e. mean age between 18 and 60 years) [36, 40–43, 45–47, 49–51, 53–55]. More than half of the studies were conducted with healthy participants (11/19) [39, 42, 43, 45, 47–49, 51–54], five studies were conducted with overweight/obese participants [36, 40, 41, 50, 55], and one study each was conducted with Diabetes Type 2 patients [44], postmenopausal women diagnosed with stage I-III breast cancer [37] and Multiple Sclerosis patients [46]. Studies used a range of tools to self-monitor sedentary behavior. Specifically, six studies used a pedometer [41, 44, 47, 49, 50, 55], four studies used a traditional/electronic logbook or a questionnaire [43, 51, 52, 55], three studies used the Jawbone Up 24 [46, 48, 53], and one study each used the Shimmer accelerometer [54], the Fitbit One [39], the Gruve [40], the Lumoback [42], the Darma Crushion [45], the Garmin Vivofit 2 [37], and the SitFIT [36]. The shortest interventions lasted 1 week [53, 54], while the longest interventions lasted 1 year [40, 45]. Twelve studies focused only on sedentary behavior [38–43, 45, 49, 50, 52, 53, 55], whereas six studies focused on the combination of sedentary behavior and physical activity [36, 37, 44, 46–48]. One study focused on the combination of sedentary behavior, physical activity and dietary behavior [51]. Twelve studies objectively measured sedentary behavior [36, 37, 39, 41, 42, 44, 45, 48–50, 53, 54], four studies subjectively measured sedentary behavior [46, 47, 51, 52], and three studies combined both methods to estimate sedentary behavior [40, 43, 55].

**Table 2:** Summary of the included intervention studies

Study characteristics		Sample characteristics		Intervention characteristics				Control	Sedentary behavior outcome
Author, year, country	Study design	Sample size	Age, gender, healthy vs clinical population	Duration	Focus	Self-monitoring	Other behavior change techniques	No intervention or other behavior change techniques	Measurement instrument used to objectively/subjectively measure total or domain-specific sedentary behavior
Arrogi, 2017, Belgium [54]	Pretest posttest control group design	*N* = 65 IG: 31 CG: 27	Mean age = 36.2 ± 10.2y 48% male Healthy population	1 week	Sedentary behavior	Self-monitoring by means of a electronic SB device: Motion sensor Shimmer (attached to the thigh) + smartphone application Information on current behavior (i.e. time spent standing or time spent sitting), daily sedentary score and sedentary index	StAPP application including information on consequences of the behavior, instructions on how to perform the behavior, feedback on performance and general encouragement	No intervention	Objectively measured total SB by means of ActivPAL3 inclinometer – attached to the thigh
Adams, 2013, USA [55]	Non-equivalent pretest posttest control group design	*N* = 64 IG: 40 CG: 24	Mean age = 58.5 ± 12.5y 0% male Obese population	6 weeks	Sedentary behavior	Self-monitoring by means of a logbook Information was reported on the number of steps (logbook and pedometer) and daily sitting time (logbook)	Information on consequences of the behavior, instructions on how to perform the behavior, feedback on performance, and goal-setting, action planning	No intervention	Objectively measured total SB by means of Actigraph GT3X accelerometer – attached to the right hip Self-reported domain-specific SB by means of a weekly sitting inventory
Ashe, 2015, Canada [39]	Pretest posttest control group design	*N* = 25 IG: 13 CG: 12	Mean age = 64.1 ± 4.6y 0% male Healthy population	6 months	Sedentary behavior	Self-monitoring by means of electronic PA device: Fitbit One (worn in the pocket) Information on daily steps, distance walked, stairs climbed, sedentary time, and low/moderate/vigorous physical activity	Information on consequences of the behavior, instructions on how to perform the behavior, goal setting, action planning, barrier identification and problem solving, graded tasks, review of behavioral goals, social support	Intervention without self-monitoring – only information on the consequences of the behavior	Objectively measured total SB by means of Actigraph GT3X accelerometer – attached to the hip
Biddle, 2015, UK [40]	Pretest posttest control group design	*N* = 187 IG: 94 CG: 93	Mean age = 32.8 ± 5.6y 31.5% male Overweight and obese population	12 months	Sedentary behavior	Self-monitoring by means of the electronic SB device: Gruve (attached to the waist) Information on time spent sedentary, in light physical activity and in moderate-to-vigorous physical activity	Information on the consequences of the behavior, goal setting, barrier identification and problem solving	Intervention without self-monitoring – only information on the consequences of the behavior	Objectively measured total SB by means of Actigraph GT3X accelerometer – attached to the right hip Self-reported total and domain-specific SB by means of the Total and Domain-Specific Sitting Questionnaire
Brakenridge, 2016, Australia [42]	Non-equivalent pretest posttest control group design	*N* = 153 IG: 66 CG: 87	Mean age = 38.9 ± 8.0y 54.0% male Healthy population	3 months	Sedentary behavior	Self-monitoring by means of the electronic SB device: Lumoback (attached to the waist) Information on sitting time, standing time, number of steps, sitting breaks, posture and sleep	Information on consequences of the behavior, instructions on how to perform the behavior, facilitate social comparison	Intervention without self-monitoring – only information on consequences of the behavior and social comparison	Objectively measured total SB and occupational SB by means of ActivPAL3 inclinometer – attached to the dominant thigh
Carr, 2013, USA [41]	Pretest posttest control group design	*N* = 40 IG: 23 CG: 17	Mean age = 44.7 ± 9.6y 10.0% male Overweight population	12 weeks	Sedentary behavior	Self-monitoring by means of an Omron pedometer (attached to the waist) Number of steps	Information on consequences of the behavior, environmental restructuring, instructions on how to perform the behavior, social support	No intervention	Objectively measured total SB and occupational SB by means of the StepWatch PA monitor – attached to the ankle
De Cocker, 2016, Belgium [43]	Multigroup pretest posttest design	*N* = 213 IG: 78 IG: 84 CG: 51	Mean age = 40.3 ± 9.1y 31.5% male Healthy population	1 month	Sedentary behavior	Self-monitoring by means of the Workforce Sitting Questionnaire Information was reported on time spent sitting on a workday and a non-workday for the last 7 days while (1) travelling to and from places, (2) being at work, (3) watching television, (4) using a computer at home (not work related), and (5) doing other leisure activities.	Information on consequences of the behavior, normative information, feedback on performance, action planning, goal setting	No intervention	Objectively measured total SB by means of the ActivPAL inclinometer – attached to the thigh Self-reported domain-specific SB by means of the Workforce Sitting Questionnaire
De Greef, 2011, Belgium [44]	Pretest posttest control group design	*N* = 92 IG: 60 CG: 32	Mean age = 62.0 ± 9.0y 69.0% male Type 2 Diabetes population	24 weeks	Sedentary behavior and physical activity	Self-monitoring by means of a pedometer^a,b^ Information on the number of steps	Information on consequences of the behavior, motivational interviewing, goal setting, problem-solving, social support	No intervention	Objectively measured total SB by means of an accelerometer^b^
Edwardson, 2018, UK [45]	Non-equivalent pretest posttest control group design	*N* = 146 IG: 77 CG: 69	Mean age = 41.2 ± 11.1y 20.0% male Healthy population	12 months	Sedentary behavior	Self-monitoring by means of an electronic SB device: Darma Cushion (placed on an chair) Information on total sitting time, and prolonged sitting time	Information on consequences of the behavior, environmental restructuring, social support, action planning, goal setting, motivational interviewing	No intervention	Objectively measured total SB and occupational SB by means of ActivPAL micro inclinometer – attached to the right thigh
Kitagawa, 2019, Japan, [53]	Multigroup pretest posttest design	*N* = 48 IG:16 IG:16 CG:16	Mean age = 38.0 ± 4.5y 0% men Healthy population	1 week	Sedentary behavior	Self-monitoring by means of an electronic PA device: Jawbone Up 24 (worn around the wrist) Information on the number of steps, total physical activity, longest activity time, longest prolonged sitting time, calorie consumption, and activity amount per time zone	Information on consequences of the behavior, feedback on performance	Intervention without self-monitoring – only information on the consequences of the behavior	Objectively measured longest prolonged sitting time by means of the Jawbone Up 24 – attached to the wrist
Klaren, 2016, USA [46]	Pretest posttest control group design	*N* = 70 IG: 33 CG: 37	Mean age = 49.9 ± 9.1y 20.0% male Population with multiple sclerose	6 months	Sedentary behavior and physical activity	Self-monitoring by means of a Yamax SW-401 digiwalker pedometer (attached to the waist) Information on the number of steps	Information on consequences of the behavior, goal setting, problem-solving, action planning, social support, social reward	No intervention	Self-reported total SB by means of the Short International Physical Activity Questionnaire
Lin, 2018, Taiwan [47]	Non-equivalent pretest posttest control group design	*N* = 101 IG: 51 CG: 50	Mean age = 49.5 (SD not reported) 47.5% male Healthy population	3 months	Sedentary behavior and physical activity	Self-monitoring by means of a Yamax SW-200 digiwalker pedometer (attached to the waist) Information on the number of steps	Information on consequences of the behavior, goal setting, barrier identification, social support, review of behavioral goals, rewards contingent on successful behavior	Intervention without self-monitoring – only information on the consequences of the behavior	Self-reported total SB by means of the Short International Physical Activity Questionnaire and self-reported occupational sedentary behavior by means of the Occupational Sitting and Physical Activity Questionnaire
Lynch, 2019, Australia [37]	Pretest posttest control group design	*N* = 83 IG:43 CG:40	Mean age = 61.6 ± 6.4y 0% men Postmenopausal women diagnosed with stage I-III breast cancer	12 weeks	Sedentary behavior and physical activity	Self-monitoring by means of a Garmin Vivofit 2 activity monitor (worn around the wrist) Information on the number of steps, distance, calories and sleep/rest time	Information on consequences of the behavior, goal setting, action planning, feedback on performance, motivational interviewing	No intervention	Objectively measured total SB by means of ActivPAL inclinometer – attached to the right thigh
Lyons, 2017, USA [48]	Pretest posttest control group design	*N* = 40 IG: 20 CG: 20	Mean age = 61.5 ± 5.6y 15.0% male Healthy population	12 weeks	Sedentary behavior and physical activity	Self-monitoring by means of an electronic PA device: Jawbone Up 24 (worn around the wrist) Information on the number of steps, calories burned and sleep	Information on consequences of the behavior, goal setting, motivational interviewing, social support, review of behavioral goals, problem-solving, self-rewards, when and where to perform the behavior, relapse prevention, stress management, time management	No intervention	Objectively measured total SB by means of the ActivPAL inclinometer – attached to the right thigh
Maylor, 2018, UK [49]	Non-equivalent pretest posttest control group design	*N* = 89 IG: 48 CG: 41	Mean age = 43.4 (40.7–45.9) 43.0% male Healthy population	8 weeks	Sedentary behavior	Self-monitoring by means of a pedometer^a,b^ Information on the number of steps	Information on consequences of the behavior, prompt practice, motivational interviewing, environmental restructuring	No intervention	Objectively measured total SB and occupational SB by means of ActivPAL micro inclinometer – attached to the right thigh
Smith, 2012, USA [50]	Pretest posttest control group design	*N* = 40 IG: 23 CG: 17	Mean age = 44,7 ± 9,3y 10.0% male Overweight population	12 weeks	Sedentary behavior	Self-monitoring by means of a Yamax SW-200 digiwalker pedometer (attached to the waist) Information on the number of steps	Environmental restructuring, feedback on performance, social support, goal setting	No intervention	Objectively measured total SB by means of the StepWatch3.0 accelerometer – attached to the ankle
Spring, 2018, USA [51]	Multigroup pretest posttest design	*N* = 128 IG: 84 CG: 44	Mean age = 40,8 ± 11,9y 23,6% male Healthy population	9 months	Sedentary behavior, physical activity and dietary behavior	Self-monitoring by means of the electronic logbook of the Make Better Choices App Information reported on leisure screen time	Feedback on performance, motivational interviewing, goal setting	No intervention	Self-reported domain-specific SB (sedentary leisure screen time) by means of the Make Better Choices app
White, 2017 UK [52]	Pretest posttest control group design	*N* = 103 IG: 52 CG: 51	Mean age = 68.3 ± 3.8y 41.0% male Healthy population	12 weeks	Sedentary behavior and physical activity	Self-monitoring by means of a tick sheet with tips on SB Information reported on the daily adherence to tips	Information on consequences of the behavior, goal setting, action planning, graded tasks, prompt practice, habit formation	No intervention	Self-reported total SB by means of the Short International Physical Activity Questionnaire and the Measure of Older adults’ Sedentary Time
Wyke, 2019, UK [36]	Pretest posttest control group design	*N* = 1113 IG: 560 CG: 553	Mean age = 45.8 ± 8.9y 100.0% male Overweight population (BMI ≥ 27 kg/m^2^)	12 weeks	Sedentary behavior and physical activity	Self-monitoring by means of an electronic SB device: SitFIT (worn in the pocket) Information on upright time, number of steps, and percentage sedentary time of awake time	Information on consequences of the behavior, goal setting, goal reviewing, action planning, social support	No intervention	Objectively measured total SB by means of the ActivPAL micro inclinometer – attached to the thigh

*N* number of participants, *IG* intervention group, *CG* control group, *y* years

^a^ Type of pedometer was not specified

^b^ Placement of self-monitoring/measurement tool was not specified

### Methodological study quality

Detailed results of the quality assessment are presented for each study in Table 3. Shortly, four studies were rated as strong [40, 44, 47, 50], five as moderate [36, 38, 41, 42, 49] and ten as weak [37, 39, 43, 45, 46, 48, 51–53, 55]. Weak ratings were frequently caused by recruitment issues, and lack of reporting on potential confounders and reasons for drop-out.

**Table 3:** Quality assessment of the included studies according to the EPHPP tool

Study	Component rating						Global rating
	Representativeness	Design	Confounders	Blinding^a^	Methods	Drop-outs	
Arrogi, 2017	Weak	Strong	Strong	Weak	Strong	Strong	Moderate
Adams, 2013	Moderate	Strong	Weak	Weak	Strong	Weak	Weak
Ashe, 2015	Weak	Strong	Weak	Moderate	Strong	Moderate	Weak
Biddle, 2015	Moderate	Strong	Strong	Weak	Strong	Moderate	Strong
Brakenridge, 2016	Moderate	Strong	Strong	Weak	Strong	Weak	Moderate
Carr, 2013	Weak	Strong	Strong	Moderate	Strong	Moderate	Moderate
De Cocker, 2016	Weak	Strong	Strong	Weak	Strong	Weak	Weak
De Greef, 2011	Moderate	Strong	Strong	Weak	Strong	Strong	Strong
Edwardson, 2018	Weak	Strong	Weak	Moderate	Strong	Moderate	Weak
Kitagawa, 2019	Weak	Strong	Strong	Moderate	Weak	Strong	Weak
Klaren, 2016	Weak	Strong	Strong	Weak	Strong	Weak	Weak
Lin, 2018	Moderate	Strong	Strong	Weak	Strong	Strong	Strong
Lynch, 2019	Weak	Strong	Weak	Weak	Strong	Strong	Weak
Lyons, 2017	Weak	Strong	Weak	Weak	Strong	Strong	Weak
Maylor, 2018	Moderate	Strong	Weak	Weak	Strong	Strong	Moderate
Smith, 2012	Moderate	Strong	Strong	Weak	Strong	Moderate	Strong
Spring, 2018	Weak	Strong	Strong	Weak	Strong	Weak	Weak
White, 2017	Weak	Strong	Weak	Moderate	Strong	Strong	Weak
Wyke, 2019	Weak	Strong	Strong	Moderate	Strong	Strong	Moderate

^**a**^ The overall risk of bias was calculated for each study based on the EPHPP guidelines (https://merst.ca/ephpp/) without taking into account the blinding score (see Methods). A strong rating was allocated to studies without weak ratings, a moderate score was allocate to studies with one weak rating, and a weak rating was allocated to studies with two or more weak ratings

### Effects of interventions including self-monitoring on total sedentary behavior

**Fig. 2:**
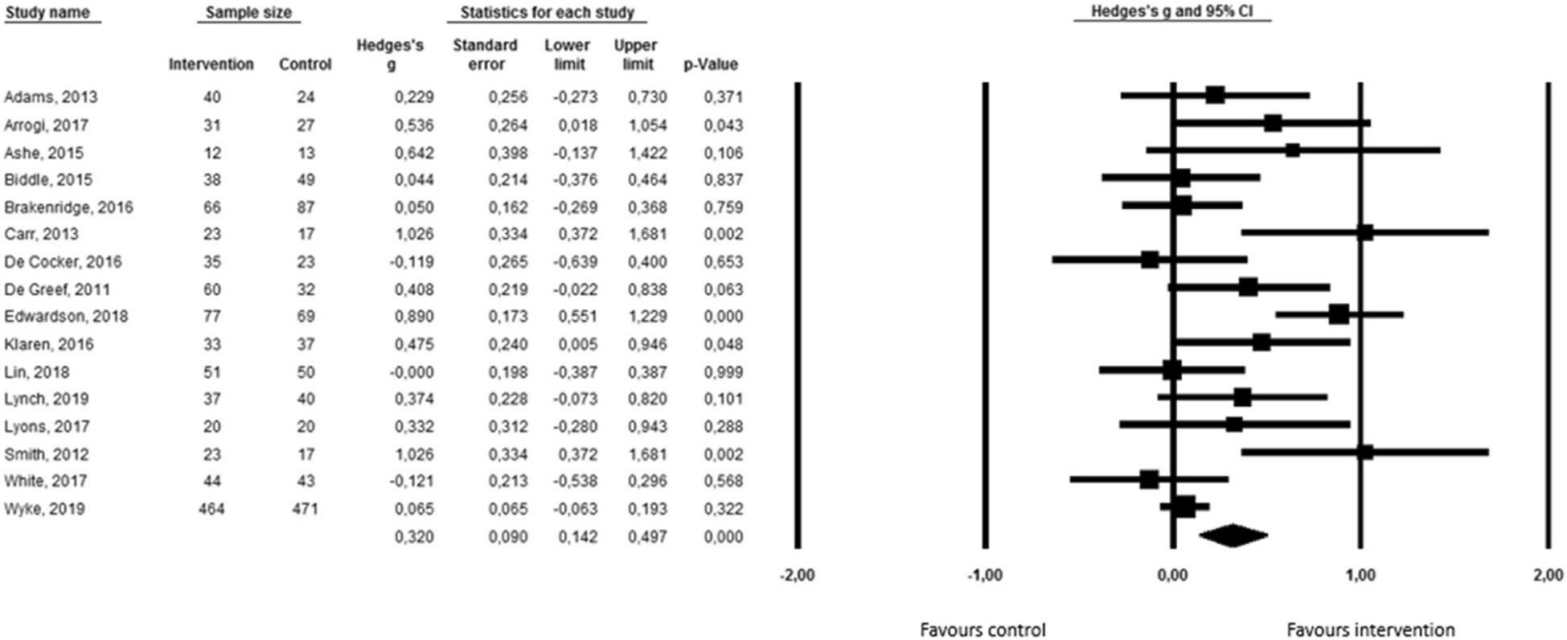
Forest plot for total sedentary behavior

Figure 2 shows the effects of 16 interventions on total sedentary behavior using a forest plot. The average effect size across all studies was significant (Hedges g = 0,32; 95% CI = 0,14-0,50; *p* = 0,001) indicating that interventions including self-monitoring as a behavior change technique have the potential to reduce total sedentary behavior in adults. The overall mean difference for total sedentary time between intervention and control groups was 34,37 min/day (95% CI = 14,48-54,25) (see Additional file 3). As such, the reduction in sedentary time is on average 34,37 min larger in the intervention group, compared to the control group. Results of the heterogeneity tests (*Q*(16) = 44,43; *p* < 0,001; *I*^*2*^ = 66,24) revealed significant heterogeneity, and thus, moderation analyses were carried out. A priori, it was decided to conduct moderation analyses on the intervention duration, the main purpose of the self-monitoring tool, the way of self-monitoring, the age group of the participants, the health status of the participants, the intervention content, and the focus of the intervention. Since all the included interventions have, however, used self-monitoring in combination with a range of other behavior change techniques, it was impossible to include intervention content as a moderator.

Results of the moderation analyses are presented in Table 4. The way of self-monitoring (i.e. subjective versus objective) was the only significant moderator (Q = 5,67; *p* = 0,02). Subgroup analyses showed that significant effects were found for interventions using objective self-monitoring tools (g = 0,40; 95% CI = 0,19-0,02; *p* < 0,001), whereas non-significant effects were found for interventions in which self-monitoring was done subjectively (g = − 0,02; 95% CI = -0,29-0,26; *p* = 0,90). The focus of the intervention was close to significance (Q = 2,88; *p* = 0,09). Interventions only focusing on sedentary behavior showed significant effects (g = 0,45; 95% CI = 0,15-0,75; *p* = 0,004), whereas interventions focusing on both sedentary behavior and physical activity did not show significant effects (g = 0,16; 95% CI = 0,001-0,31; *p* = 0,11). The main purpose of the self-monitoring tool (Q = 1,95; p = 0,16), the intervention duration (Q = 1,93; *p* = 0,17), the age group of the participants (Q = 0,17; *p* = 0,68), and the health status of the participants (Q = 0,03; *p* = 0,86) did not significantly moderate the intervention effects.

**Table 4:** Moderation analyses for total sedentary behavior

Moderator	Number of studies	Combined sample size	Hedges’ g	95% CI	Q	*P*
Intervention length					1,93	0,17
Short (≤ 12 weeks) [36, 38, 41–43, 47, 48, 50, 52, 55]	11	1653	0,23	0,04 – 0,41		
Long (> 12 weeks) [39, 40, 44–46]	5	440	0,49	0,17 – 0,81		
Main purpose of self-monitoring tool					1,95	0,16
To measure physical activity [37, 39, 41, 44, 46–48, 50, 55]	9	549	0,43	0,22 – 0,65		
To measure sedentary behavior [36, 38, 40, 42, 43, 45, 52]	7	1524	0,19	-0,07 – 0,45		
Way of self-monitoring					5,67	0,02
Subjective self-monitoring [43, 52, 55]	3	209	-0,02	-0,29 – 0,26		
Objective self-monitoring [36–42, 44–48, 50]	13	1864	0,40	0,19– 0,60		
Age group					0,17	0,68
Adults (mean age: 18–60 years) [36, 38, 40–43, 45–47, 50, 55]	11	1752	0,34	0,11 – 0,57		
Older adults (mean age > 60 years) [37, 39, 44, 48, 52]	5	321	0,27	0,02 – 0,52		
Health status					0,03	0,86
Healthy participants [38, 39, 42, 43, 45, 47, 48, 50, 52]	9	708	0,33	0,03 – 0,63		
Participants with overweight/obesity or another clinical condition [36, 40, 41, 44, 55]	7	1365	0,30	0,08 – 0,52		
Focus of the intervention					2,88	0,09
Only sedentary behavior	9	671	0,45	0,15 – 0,75		
Sedentary behavior and physical activity^a^	7	1402	0,16	0,001 – 0,31		

Hedges’ g (random effects); *CI* confidence interval, *Q* homogeneity statistic (mixed effects), ^a^One study focused on sedentary behavior, physical activity and dietary behavior

Funnel plot and Egger’s Test (t(16) = 2,24; *p* = 0,05) indicated that publication bias was unlikely to have influenced the results (see Additional file 4). Sensitivity analyses showed that the effect sizes largely remained within the 95% confidence interval after removing the study of Wyke et al. (g = 0,35; 95% CI = 0,16-0,55; *p* < 0,001), after removing low quality studies (g = 0,29; 95% CI = 0,06-0,52; *p* = 0,012), after removing cluster-randomized controlled trials (g = 0,32; 95% CI = 0,12-0,52; *p* = 0,001), and after removing studies using subjective measurement instruments (g = 0,40; 95% CI = 0,17-0,62; *p* = 0,001).

### Effects of interventions including self-monitoring on domain-specific sedentary behavior

**Fig. 3:**
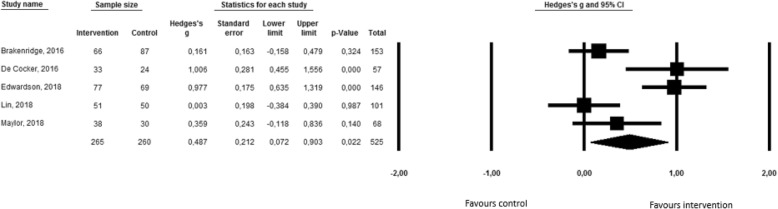
Forest plot for occupational sedentary behavior

Six of the included interventions assessed the influence on domain-specific sedentary behavior. Of these, one study examined the effect on leisure screen time [51], four on occupational sitting time [42, 45, 47, 49], and one on different domains of sedentary behavior (i.e. at work, for transport, computer time, television time and other leisure time) [43]. Given the lack of results on transport-related sedentary behavior and leisure time sedentary behavior, a meta-analysis was only conducted on occupational sedentary behavior. Results of this meta-analysis, which are presented by means of the forest plot in Fig. 3, show that occupational sedentary behavior significantly reduced after the intervention including self-monitoring (Hedge’s g = 0,49; 95% CI = 0,07-0,90; *p* = 0,02). Although significant heterogeneity was detected (Q(5) = 21,44; *p* < 0,001; I^2^ = 81,34), no moderation analyses were conducted due to the limited number of studies (i.e. at least three studies were required per category to perform the moderation analyses) [56].

Funnel plot and Egger’s Test (t(5) = 0,45; *p* = 0,68) indicated that publication bias was unlikely to have influenced the results (see Additional file 4). Sensitivity analyses were not performed as there were no outliers, no studies with extreme large sample size, and only three studies with moderate or strong quality. Sample sizes of the latter three studies were too small to conduct meaningful analyses [56].

### Effects of interventions including self-monitoring on the number of breaks in sedentary behavior

**Fig. 4:**
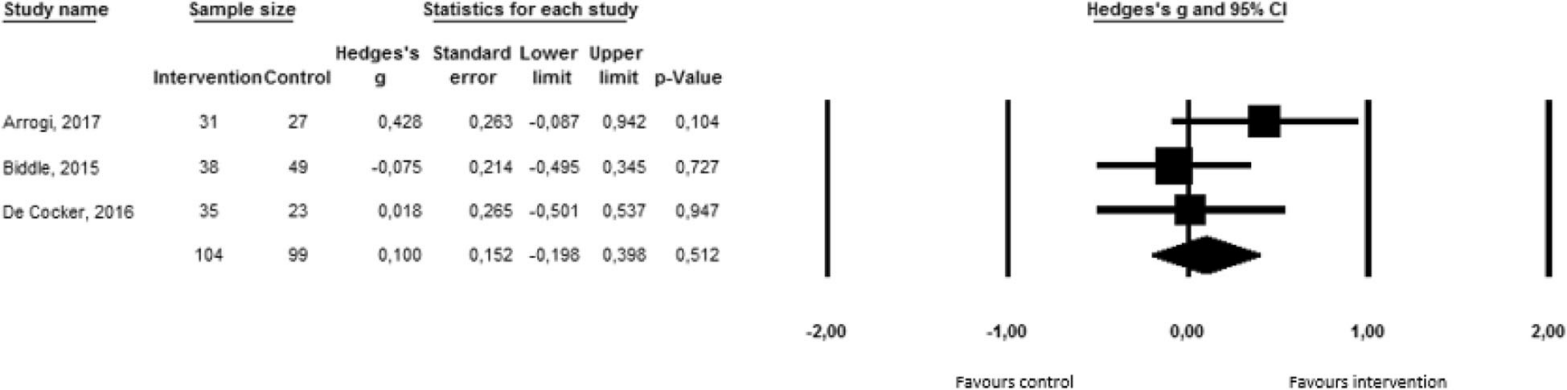
Forest plot for the number of breaks in sedentary behavior

Only four of the included studies examined the effect of an intervention using self-monitoring on the number of breaks in sedentary behavior. The effect size of one of the studies [49] exceeded the outlier threshold of three standard deviations above the average effect size. This study was thus removed from the analyses. Figure 4 presents the effects of the remaining three studies, as well as the average effect size by means of a forest plot. The average effect size was not significant (Hedge’s g = 0,10; 95%CI = -0,18-0,37; *p* = 0,50), meaning that the existing interventions including self-monitoring were not able to increase the number of breaks in sedentary behavior. No significant heterogeneity was found. Funnel plot and Egger’s Test (t(3) = 0,90; *p* = 0,53) indicated that publication bias was unlikely to have influenced the results (see Additional file 4). Again, sensitivity analyses were not performed due to the low number of studies.

## Discussion

Results of the current meta-analyses suggest that interventions including self-monitoring have the potential to reduce sedentary behavior in adults, and may thus play a critical role in promoting public health. If future studies can prove that the positive effects on sedentary behavior are attributable to self-monitoring, this finding will be in line with the results of a recent review by Gardner et al., which identified self-monitoring as one of the most promising behavior change techniques in sedentary behavior reduction interventions [15]. Given that the effect sizes in the current meta-analyses were rather small, more research should be conducted to examine 1) if the reduction in sedentary time is clinically relevant, and 2) how the reduction in sedentary time might be enhanced.

Specifically, our results indicated that both total sedentary behavior, and occupational sedentary behavior significantly reduced after implementing a self-monitoring based intervention. These reductions were, however, only significant if an objective self-monitoring tool was used. This moderating effect is not surprising, and can be explained by the fact that sedentary behavior is a largely subconscious behavior, and thus difficult – and time-consuming – to self-report [57]. Using objective self-monitoring tools may reduce the burden and increase the adherence to the intervention, and thus result in better achievements towards the behavioral goals [35].

Next, our results suggested that interventions only focusing on the reduction of sedentary behavior are more effective in comparison to interventions focusing on both the reduction of sedentary behavior and the increase of physical activity. This finding corresponds with the results of the review of Prince et al. [58]. In their review, Prince et al. showed that interventions only targeting sedentary behavior have the largest impact on sedentary behavior (i.e. a mean reduction of 91 min per day). Interventions targeting both sedentary behavior and physical activity resulted in a mean reduction of 35 min sedentary behavior per day [58]. This difference in effect size might be explained by the fact that participants of interventions targeting both sedentary behavior and physical activity are more likely to focus on physical activity due to 1) the clearer guidelines for physical activity compared to sedentary behavior, 2) the better known negative health consequences of too little physical activity compared to sedentary behavior, and 3) the fact that physical inactivity is still considered a synonym for sedentary behavior in the general population [59]. This might be important, as existing self-monitoring devices often combine the information on sedentary behavior and physical activity. For example, the device used in the study of Wyke et al. (i.e. the SitFIT) provided information on the number of steps and on the time spent sedentary [36]. Participants of this study seemed to mostly look at the number of steps taken, and paid less attention to the amount spent sitting. They indicated that they were more familiar with the step counts, and they considered the sitting time feedback as complicated [36]. Based on these results, it could be concluded that in order to reduce sedentary time 1) self-monitoring devices should be developed only focusing on sedentary behavior, and 2) information on sitting time should be optimized. However, as the aim of most behavioral interventions is to maximize health benefits, it might be useful to target both sedentary behavior and physical activity. Both behaviors have the ability to induce beneficial health effects, and are part of the 24 h movement continuum [60]. As such, changing one behavior also affects another behavior [60].

Other potential moderators that were tested (i.e. intervention duration, main purpose of the self-monitoring tool, age group and health status) did not account for differences between effect sizes. This is in contrast with our expectations, as we predicted larger effects in longer interventions, and in interventions of which the self-monitoring tool was specifically developed to reduce sedentary behavior. Longer interventions were expected to yield better results due to the dose-response relationship. Nevertheless, based on the current results it is unclear if the dose was higher in longer interventions, as information on frequency, and intensity was incomplete [61], but other differences in intervention content might have played a role as well. Self-monitoring tools specifically developed to reduce sedentary behavior were expected to generate larger gains due to the fact that they are more appropriate to bring sedentary behavior into conscious awareness. More research is needed to understand why this was not the case. It might, however, be that existing physical activity devices include much more behavior change strategies (e.g. providing feedback, social support etc.) compared to the existing sedentary behavior devices, which are still in infancy [35]. Given that several of the included moderators were not significant, part of the heterogeneity remains unexplained, and should thus be examined in future studies. One of the main factors, that was not examined due to the lack of studies, and that might have caused the unexplained heterogeneity are the other behavior changes techniques included in the interventions. For instance, some of the interventions applied environmental restructuring, by for example, implementing standing desks at work [45]. Implementing standing desks has been shown to be successful in reducing sedentary behavior [62, 63]. As such, these interventions might have yielded larger effects.

Finally, it should be acknowledged that the few studies that analyzed the effect on the number of breaks were not able to detect significant increases. This is disappointing as previous research has emphasized that interrupting bouts of sedentary behavior with light-intensity physical activity might yield beneficial health effects [64]. More research is needed to understand the underlying reasons for the non-significant effects, although the lack of information on breaks of the existing self-monitoring tools might have played a crucial role [35].

### Strengths and limitations

The main strength of this study is that this is the first study, to our knowledge, to synthesize and statistically analyze the existing evidence regarding the effectiveness of interventions that use self-monitoring as a behavioral change technique to reduce sedentary behavior in adults. Moreover, the study used a comprehensive search strategy across multiple databases to identify all relevant studies, including grey literature. Both the screening of eligible studies, and the quality assessment has been conducted by two independent reviewers, and the quality score has been used to conduct sensitivity analyses.

The main limitation of the current study is the fact that self-monitoring was never implemented as a stand-alone strategy in the existing interventions. All the included interventions, however, comprised a range of behavior change techniques, and thus it remains uncertain if the effects are attributable to self-monitoring in itself, or if (a combination with) other behavior change techniques induced the behavioral change. Future studies should try to disentangle the combinations of behavior change techniques in order to gain insight into the working mechanism of the separate behavior change techniques as well as their interactions. By doing so, effective interventions of minimal costs and efforts can be created. Secondly, several of the included studies were pilot studies, and thus, performed with small sample sizes. Studies with small sample sizes are often underpowered to detect small changes in sedentary behavior. This might have led to an underestimation of the actual effect size. Thirdly, many of the included studies had a low methodological quality, due to weak ratings on selection bias, confounders and withdrawals. Although these weak ratings might be due to a lack or reporting, this might have affected the results. More high quality studies are needed with larger samples and objective measures in future research. Finally, we were only able to analyze short-term effects, as the majority of the included interventions did not report long-term effects. As such, it remains unclear whether intervention effects will be sustained over time. Insight into the long-term effectiveness is of great importance to evaluate the actual public health impact of self-monitoring based sedentary behavior interventions [65].

## Conclusion

Results of the current meta-analysis – which included 17 intervention studies – suggest that interventions including self-monitoring show promise to reduce short-term sedentary behavior in adults. Important to note, however, is that all the included interventions have employed multiple behavior change techniques, whereby it is impossible to determine if the beneficial effects on sedentary behavior are attributable to self-monitoring in itself, or to (a combination with) other behavior change techniques. Moderation analyses indicated that the reductions in sedentary behavior are larger if an objective self-monitoring tool was used, and if the intervention only focuses on sedentary behavior. Still, from a public health point of view, combined physical activity and sedentary behavior interventions would be preferred but more research is needed to effectively integrate both behaviors in intervention strategies. If future studies can confirm that previous mentioned positive effects are long-lasting, the development and implementation of sedentary behavior interventions including self-monitoring as a key behavior change technique should be encouraged.

## Additional files

Additional file 1: PRISMA checklist. (DOC 63 kb)

Additional file 2: Protocol of the meta-analysis. (DOCX 45 kb)

Additional file 3: Difference in means for total sedentary time between the intervention group and the control group. (DOCX 117 kb)

Additional file 4: Publication bias. (DOCX 111 kb)

## Data Availability

The dataset is available from the corresponding author on reasonable request.

## Abbreviations

CG: Control group
CI: Confidence interval
EPHPP: Effective Public Health Practice Project
IG: Intervention group
N: Number of participants
PRISMA: Preferred reporting items for systematic reviews and meta-analysis
