# Supplementary material for: Effectiveness of interventions using self-monitoring to reduce sedentary behavior in adults: a systematic review and meta-analysis
Source: Int J Behav Nutr Phys Act. 2019 Aug 13;16:63. doi: 10.1186/s12966-019-0824-3 (PMC6693254; doi:10.1186/s12966-019-0824-3)
Supplement: Supplementary file 2 — Protocol of the meta-analysis. (DOCX 45 kb) [file 12966_2019_824_MOESM2_ESM.docx]

# Study Protocol

# Self-monitoring based interventions to reduce sedentary behavior in adults and older adults: a systematic review and meta-analysis

1. **Objective**

To investigate the efficacy of self-monitoring based interventions aimed at the reduction of sedentary behavior in adults and older adults.

To identify factors moderating the observed efficacy:

- Intervention content (only self-monitoring [and general information on the link between behavior and health outcome] vs combined with other behavioral change techniques)
- Intervention length
- Self-monitoring tool (sedentary behavior tool vs physical activity tool, and self-reported vs electronic device)
- Age group of the participants (adults [i.e. mean age < 65 years] vs older adults [i.e. mean age ≥ 65 years)
- Study quality

# Criteria for considering studies for this review

# Type of studies

Only controlled intervention trials, in which an effect size can be calculated on sedentary behavior, and with at least 10 participants in each arm, were included:

1. RCTs (randomly assigning individuals to intervention and comparison groups),
2. Cluster-RCTs (randomly assigning groups to intervention and comparison groups)
3. Non-randomised controlled trials (non-RCT; assigning subjects using methods that are not random, e.g. (partly) based on preference).

Within subject studies (e.g. one group pretest posttest design) have no control group, and where thus not eligible in the current review.

Studies which reported the same outcomes from identical or overlapping samples in several articles, were only included once (most complete sample). If different outcomes were reported on the same intervention in more than one article, these were grouped in one study.

# Type of participants

Studies with participants with a mean age above 18 years were included. Studies could be conducted with either a general population or with patient groups, as long as the intervention type met the inclusion criteria (see 2.3). Patient groups were not excluded as the intervention is not cognitive based, and sedentary behavior does not interfere with the majority of clinical syndromes.

If the overall mean age was not reported and mean age of groups was reported, the overall mean was calculated using the following formula ((M1*N1) + (M2*N2)) /(N1+N2).

# Type of intervention

Evidence on the working strategies of interventions to reduce adults and older adults’ sedentary behavior is still limited [5, 10]. Existing sedentary behavior interventions have – just like physical activity – been largely informed by social-cognitive models of behavioral change (e.g. Theory of Planned Behavior) [1]. However, most of these models are based on an expectancy-value framework in which behavior is determined by expected outcomes and the value that is placed on them [2]. As such, these models inadequately capture processes underlying unintentional and habit-like behavior. Given that we believe that a large part of sedentary behavior is habitual (i.e. it does not involve reflective thinking, and occurs often out of the focus of attention) [3], specific strategies are needed to control sedentary behavior. Chief amongst these strategies is to raise the conscious awareness of the habitual behavior, which might be achieved by means of self-monitoring.

As such, interventions are included in the current review if they are:

1. Self-monitoring based i.e. if the participants were asked to keep a record of a specified behavior as a method of changing behavior [4], and if the participants has access to the recorded data of behavioral performance [5]. Interventions in which the participants were asked to wear a blinded monitor (e.g. Activpal) were hence not included.

Self-monitoring should be an explicitly stated intervention component, as opposed to occurring as part of completing measures for research purposes. This could for example take the form of 1) a(n) (online) diary, 2) completing a questionnaire about their behavior, in terms of type, frequency, duration and/or intensity, and/or 3) the use of an electronic device [4].

1. Aimed to reduce sedentary behavior (i.e. any waking activity characterized by an energy expenditure ≤ 1.5 metabolic equivalents (METs) performed in a sitting, reclining, or lying posture [6]); Both interventions aimed at

- the reduction of total sedentary behavior,
- the reduction of domain-specific sedentary behavior (e.g. screen time, occupational sitting time, etc.)
- the reduction of prolonged sedentary bouts
- or at increasing the number of sedentary behavior interruptions

were eligible in the current review. Interventions in which the primary aim was to increase physical activity were excluded, as previous research has indicated that these interventions revealed no, or only small effects on sedentary behavior [7].

Self-monitoring has been identified in the review of Gardner as a promising intervention strategy to reduce sedentary behavior in adults and older adults [8]. However, it should be noted that the majority of the studies included in the review used self-monitoring in relation to physical activity (e.g. self-monitoring bouts of physical activity), rather than sedentary behavior. This might not be surprising, as at the moment of the review, self-monitoring sedentary behavior was only possible via the traditional paper-based diaries or online recoding forms. More recently, bodily worn electronic devices, such as the SitFit, Lumboback, and the Jawbone Up or the Vivofit, have emerged as a result of technological advances. These electronic devices have reduced the burden of traditional methods, which might have resulted in improved adherence, and in turn, greater achievement towards behavior goals [9].

Given that bodily worn electronic devices aimed at the reduction of sedentary behavior are relatively new, we expect that many of the identified sedentary behavior interventions will use a monitor of which the main purpose is to track physical activity (e.g. pedometer, or physical activity tracker). However, we decided to include this kind of interventions, and to conduct moderation analyses to examine the differences in effect size depending on the main purpose of the self-monitoring tool. In order to assess the main purpose of the self-monitoring tools, websites from manufacturers and the scoping review of Sander et al. will be used [10].

Comparison intervention: Control conditions may consist of no intervention, or an intervention of a different type (e.g. website, educational sessions, environmental changes). As such, all interventions in which self-monitoring was not used as a BCT, are eligible. Control conditions that use the same intervention in content but in a different intensity or duration, are not accepted, given that the aim of this study is to test if self-monitoring is an effective BCT in sedentary behavior interventions.

# Type of outcome measure

Studies will be included if one of the outcomes is sedentary behavior. Studies that only reported symptoms or health outcomes and no effects on behavior are not included, as well as studies only reporting effects on physical (in)activity.

Both studies with self-reported measured sedentary behavior (e.g. by means of questionnaires, diaries), and/or objectively measured sedentary behavior (e.g. by means of accelerometers, inclinometers) are included in the current meta-analyses. If only one domain of sedentary behavior – as supposed by Owen et al. [11] – is measured, the study will still be included in the review. If the effect on more than one domain of sedentary behavior is presented, an aggregated effect score will be calculated.

Three different meta-analyses will be conducted:

1. Total sedentary behavior
2. Domain-specific sedentary behavior
3. Number of breaks in sedentary behavior

# Search methods for identification of studies

**3.1. Database search**

Published studies were identified using the electronic databases Pubmed, Embase, Web of Science and The Cochrane Library. References and citation lists of papers and published reviews were searched. The search was limited to articles published in English between the beginning of 2000 and October 2018.

The search strategy was developed using the PICO (population, intervention, comparison, outcome) acronym. The population of interest was adults and older adults, the intervention was based on self-monitoring and (one of) the outcome(s) was sedentary behavior.

Details on the search strategy are presented below:

("intervention" OR "trial" OR "effectiveness" OR "efficacy")

AND

("sedentary behavior" OR "sedentary behaviour" OR "sedentary behaviors" OR "sedentary behaviours" OR "sedentary time" OR "sedentary lifestyle" OR "sitting time" OR "TV time" OR "TV viewing" OR "watching TV" OR "computer time" OR "computer use" OR "screen time" OR "sedentary activity" OR "sedentary activities" OR "driving" OR "passive transport" OR "car use" OR “motor transport” OR “gaming”)

AND

(adult OR individuals OR adults OR elderly OR aged OR "older people" OR seniors OR senior OR workers OR employees OR men OR women OR patients OR survivors)

Although the included interventions need to use self-monitoring as a technique to change sedentary behavior, self-monitoring was not included in the search strategy, as many studies did not use the term self-monitoring.

**3.2. Grey literature search**

Given that McAuley and colleagues have shown that the exclusion of grey literature from meta-analyses can lead to exaggerated estimates of intervention effectiveness [12], grey literature (i.e. documents with scientifically valuable information which are not published in the scientific literature) was searched – as recommended in the current Cochrane Library guidelines – using Google, and unpublished trials were sought using [http://www.controlled‐trials.com](http://www.controlled-trials.com/), and [www.clinicaltrials.gov](http://www.clinicaltrials.gov).

# Methods of the review

**4.1. Study selection**

Initial screening based on title and abstract will be performed by the first author (SC). Full texts after this first selection will be screened by two reviewers (SC & DVD) for inclusion in the review in accordance with the inclusion and exclusion criteria set in the study protocol. Consensus was used to resolve disagreement regarding inclusion of the studies. When doubt regarding the inclusion of a study persisted, a third reviewer (ADS) will be consulted.

**4.2. Data extraction**

*Study characteristics – including potential moderator variables*

Data extraction was done by SC and DVD independently using a standardized form and included: study characteristics (e.g. country, year, study design); participant characteristics (e.g. sample size, age, gender); intervention characteristics (e.g. setting, duration, content); control characteristics; and sedentary behavior measure (e.g. objective vs subjective; total sedentary behavior vs domain-specific sedentary behavior). Consensus was used to resolve disagreement regarding the coding categories. If consensus could not be reached, inconsistencies were discussed with a third reviewer (ADS).

Source characteristics:

- First author
- Country of publication (1^st^ author)
- Publication year

Study design:

- True experimental design (pretest posttest control group design, two group pretest posttest design, multigroup pretest posttest design, posttest only control group design)
- Quasi-experimental design (non-equivalent pretest posttest control group design, posttest only control group design)

Sample characteristics:

- Sample size: intervention group, control group
- Healthy vs clinical sample
- Mean age ± SD (note: if the overall mean age was not reported, but the mean age of groups was reported, the overall mean age was calculated ((M1*N1)+(M2*N2))/(N1+N2))
- Percentage of males (note: if the overall percentage of males was not reported, but the percentages of males per group was reported, the overall percentage of males was calculated ((M1*N1)+(M2*N2))/(N1+N2))

Intervention characteristics:

- Intervention setting: community, worksite, home, health care
- Intervention length
- Intervention content:
- Self-monitoring component (self-reported vs electronic device and sedentary behavior tool vs physical activity tool)
- Other intervention components (including accompanying BCTs)

Control characteristics:

- Control content
- No intervention vs intervention without self-monitoring (note: if there was an intervention without self-monitoring, the intervention components should be described together with the accompanying BCTs)

Sedentary behavior measure(s):

- Objectively measured vs self-reported
- Measurement instrument
- Total vs domain-specific; if domain-specific, information will be provided on the included domains

*Sedentary behavior outcome data – used for meta-analysis*

For the meta-analyses, unadjusted sedentary behavior outcome data were extracted in the form of means and standard deviations of each group for both pre and post assessment (or mean changes and SD differences). If no unadjusted sedentary behavior outcome data were reported, authors were contacted to provide the unadjusted sedentary behavior outcomes. If authors did not respond, the reported adjusted values were extracted. When assessments were conducted at interim time points, only post-test at latest end-point of intervention was included. Follow-up assessments, after the intervention had ended, were not considered. For studies which used multiple intervention groups – all including self-monitoring – within one study (e.g. several similar intervention groups and one control condition), the multiple groups were combined using the following formula’s (Higgings and Green, 2011):

|  | Group 1 | Group 2 | Combined groups |
| --- | --- | --- | --- |
| Sample size | N1 | N2 | N1 + N2 |
| Mean | M1 | M2 | (N1M1 + N2M2) / (N1+N2) |
| SD | SD1 | SD2 | √ (((N1-1)SD1² + (N2-1)SD2² +((N1N2)/(N1+N2)) (M1²+M2²-2M1M2)) / N1+N2 -1) |

If a study contains multiple intervention groups, but only one of the intervention groups includes self-monitoring, only means and standard deviations of the self-monitoring based intervention will be extracted.

*Methodological quality*

Methodological study quality was assessed using the Effective Public Health Practice Project (EPHPP) Quality Assessment Tool for quantitative studies (https://merst.ca/ephpp/). Studies were independently reviewed by two researchers (SC and LP) and disagreements were resolved through discussion. Each of the following aspects were rated as weak, moderate or strong, in the EPHPP:

- Selection bias
- Study design
- Confounders
- Blinding
- Data collection methods
- Withdrawals and drop-outs
- Intervention integrity
- Analyses

Based on the separate ratings, an overall risk of bias was assigned to each study. The impact of excluding low quality studies was assessed by sensitivity analyses (see below).

**4.3. Data analyses**

*Meta-analyses*

Meta-analyses were conducted using Comprehensive Meta-analyses (CMA) software version … (Biostat Inc., Englewood, NJ, USA). For each study an effect size was calculated with Hedges’ formula correcting for small samples (Hedges, 1981). By calculated Hedge’s g, all effect sizes were transformed to a common metric, which enables us to include different outcome measures in the same analysis [13]. Random effects models were used for the meta-analyses, as the true effects in the studies are assumed to have been sampled from a distribution of true effects. The random effects model estimated the mean of a distribution of effects. This method is generally used when studies are gathered from the published literature [13]. Findings of the meta-analysis were presented using a forest plot.

*Test of heterogeneity and moderation analyses*

The existence of heterogeneity was assessed using the Cochrane’s Q test, and the I^2^ statistics. A Q-value with a significance of p ≤ 0.05 was considered significant heterogeneity, while for the I^2^, 25% was considered low, 50% was considered moderate, and 75% was considered high. If high heterogeneity was present, moderator analyses were conducted to test whether the heterogeneity can be explained by differences in intervention content, intervention length, self-monitoring tool or age (see above).

*Sensitivity analyses*

Sensitivity analyses were carried out to test the ways in which the main findings were changed by varying the methodological approach. Concretely, main analyses were repeated for randomized controlled trials only, and for moderate and strong quality studies only.

*Publication bias check*

The presence of publication bias was assessed using a funnel plot and Egger’s regression test.

**References**

1. Gardner B, Smith L, Lorencatto F, Hamer M, Biddle SJ: **How to reduce sitting time? A review of behaviour change strategies used in sedentary behaviour reduction interventions among adults.** *Health psychology review* 2016, **10:**89-112.

2. Welk GJ: **The youth physical activity promotion model: a conceptual bridge between theory and practice.** *Quest* 1999, **51:**5-23.

3. Conroy DE, Maher JP, Elavsky S, Hyde AL, Doerksen SE: **Sedentary behavior as a daily process regulated by habits and intentions.** *Health Psychology* 2013, **32:**1149.

4. Michie S, Ashford S, Sniehotta FF, Dombrowski SU, Bishop A, French DP: **A refined taxonomy of behaviour change techniques to help people change their physical activity and healthy eating behaviours: the CALO-RE taxonomy.** *Psychology & health* 2011, **26:**1479-1498.

5. Michie S, Johnston M, Francis J, Hardeman W, Eccles M: **From theory to intervention: mapping theoretically derived behavioural determinants to behaviour change techniques.** *Applied psychology* 2008, **57:**660-680.

6. Tremblay MS, Aubert S, Barnes JD, Saunders TJ, Carson V, Latimer-Cheung AE, Chastin SF, Altenburg TM, Chinapaw MJ: **Sedentary Behavior Research Network (SBRN)–Terminology Consensus Project process and outcome.** *International Journal of Behavioral Nutrition and Physical Activity* 2017, **14:**75.

7. Martin A, Fitzsimons C, Jepson R, Saunders DH, van der Ploeg HP, Teixeira PJ, Gray CM, Mutrie N: **Interventions with potential to reduce sedentary time in adults: systematic review and meta-analysis.** *Br J Sports Med* 2015, **49:**1056-1063.

8. Gardner B, Smith L, Lorencatto F, Hamer M, Biddle SJ: **How to reduce sitting time? A review of behaviour change strategies used in sedentary behaviour reduction interventions among adults.** *Health Psychol Rev* 2016, **10:**89-112.

9. Turner-McGrievy GM, Beets MW, Moore JB, Kaczynski AT, Barr-Anderson DJ, Tate DF: **Comparison of traditional versus mobile app self-monitoring of physical activity and dietary intake among overweight adults participating in an mHealth weight loss program.** *Journal of the American Medical Informatics Association* 2013, **20:**513-518.

10. Sanders JP, Loveday A, Pearson N, Edwardson C, Yates T, Biddle SJ, Esliger DW: **Devices for self-monitoring sedentary time or physical activity: a scoping review.** *Journal of medical Internet research* 2016, **18**.

11. Owen N, Sugiyama T, Eakin EE, Gardiner PA, Tremblay MS, Sallis JF: **Adults' sedentary behavior determinants and interventions.** *Am J Prev Med* 2011, **41:**189-196.

12. McAuley L, Tugwell P, Moher D: **Does the inclusion of grey literature influence estimates of intervention effectiveness reported in meta-analyses?** *The Lancet* 2000, **356:**1228-1231.

13. Borenstein M, Hedges LV, Higgins JP, Rothstein HR: *Introduction to meta-analysis.* John Wiley & Sons; 2011.
