# Supplementary material for: Effectiveness of interventions using self-monitoring to reduce sedentary behavior in adults: a systematic review and meta-analysis
Source: Int J Behav Nutr Phys Act. 2019 Aug 13;16:63. doi: 10.1186/s12966-019-0824-3 (PMC6693254; doi:10.1186/s12966-019-0824-3)
Supplement: Supplementary file 3 — Difference in means for total sedentary time between the intervention group and the control group. (DOCX 117 kb) [file 12966_2019_824_MOESM3_ESM.docx]

# S3: Difference in means (min/day)

Results of fourteen studies were included in the current model. Two studies could not be included as no absolute information on the total time spent sedentary was provided.


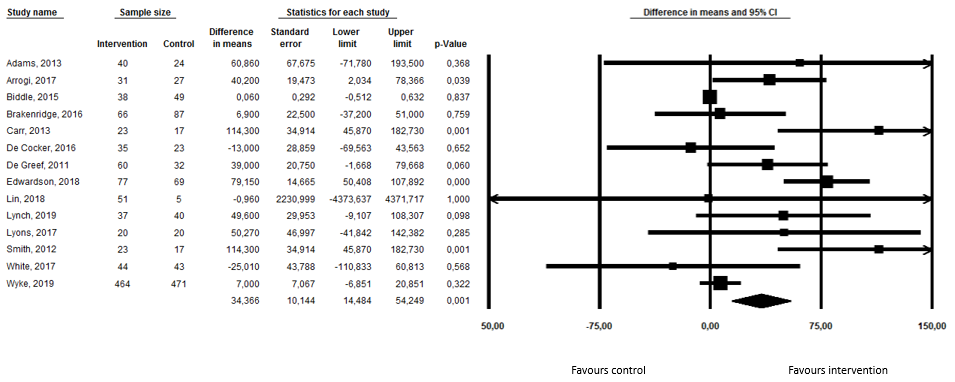


Figure 1: Forest plot for total sedentary behavior (difference in means)
