# Supplementary material for: Effectiveness of interventions using self-monitoring to reduce sedentary behavior in adults: a systematic review and meta-analysis
Source: Int J Behav Nutr Phys Act. 2019 Aug 13;16:63. doi: 10.1186/s12966-019-0824-3 (PMC6693254; doi:10.1186/s12966-019-0824-3)

# S4: Publication bias check

1.
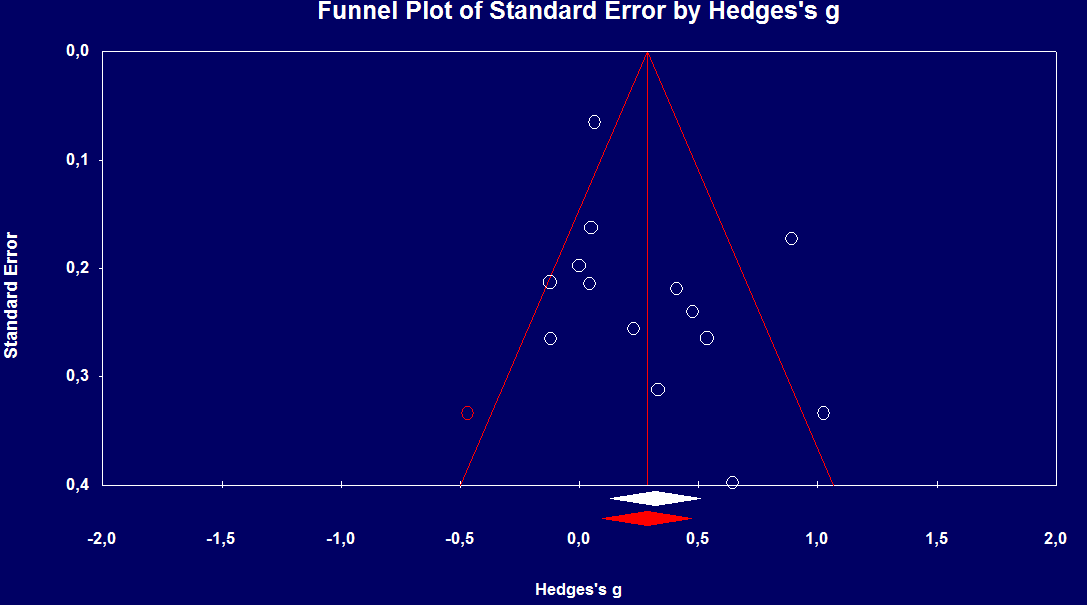
Total sedentary behavior
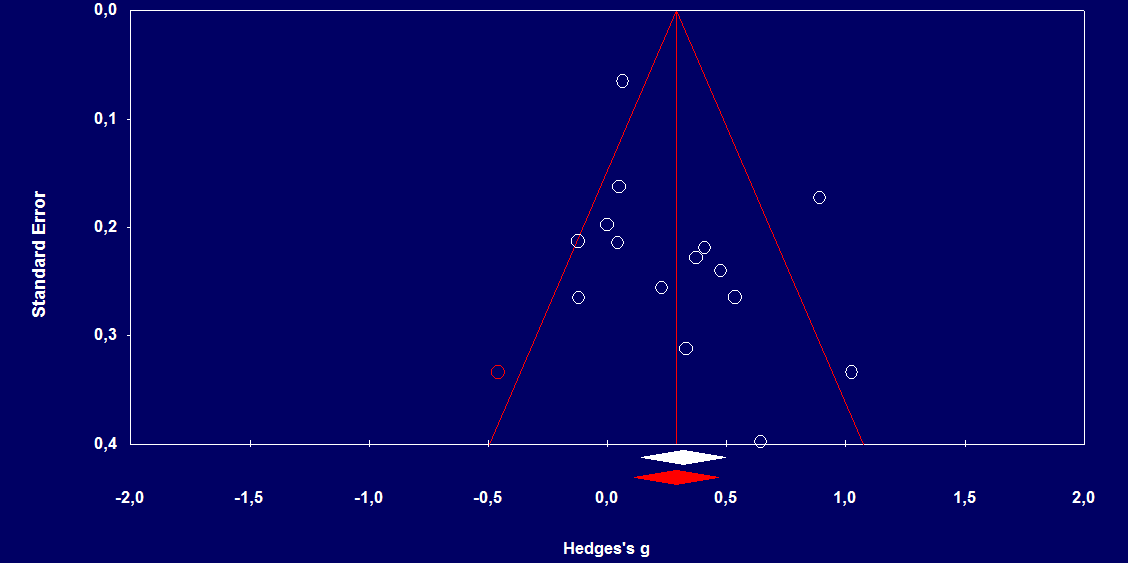

2.
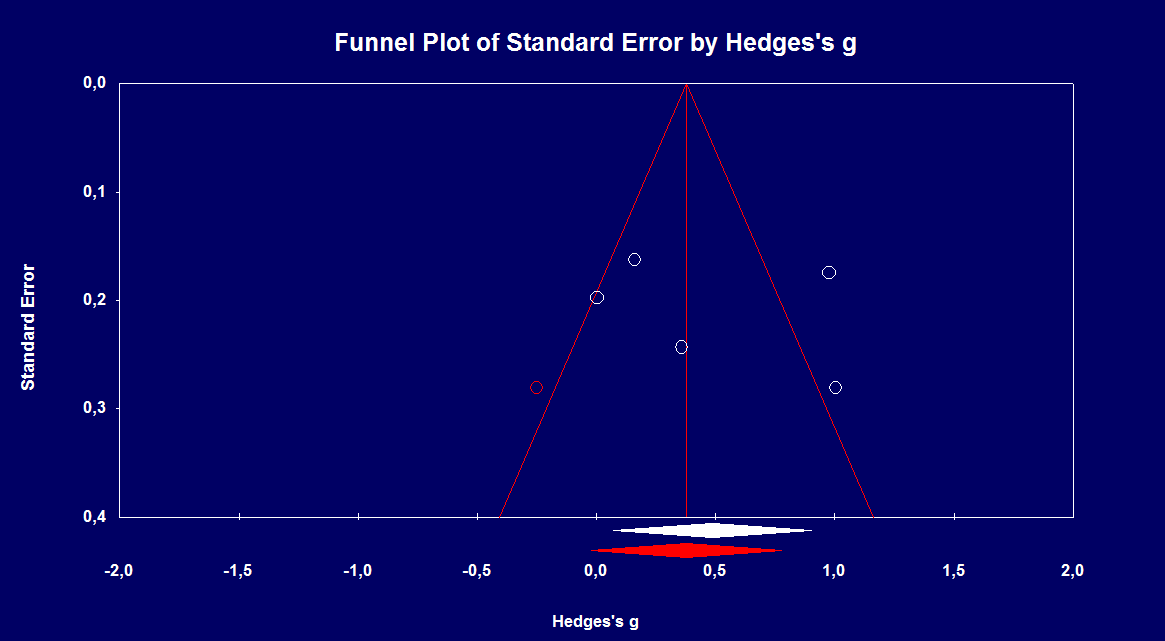
Domain-specific sedentary behavior
3. Number of breaks


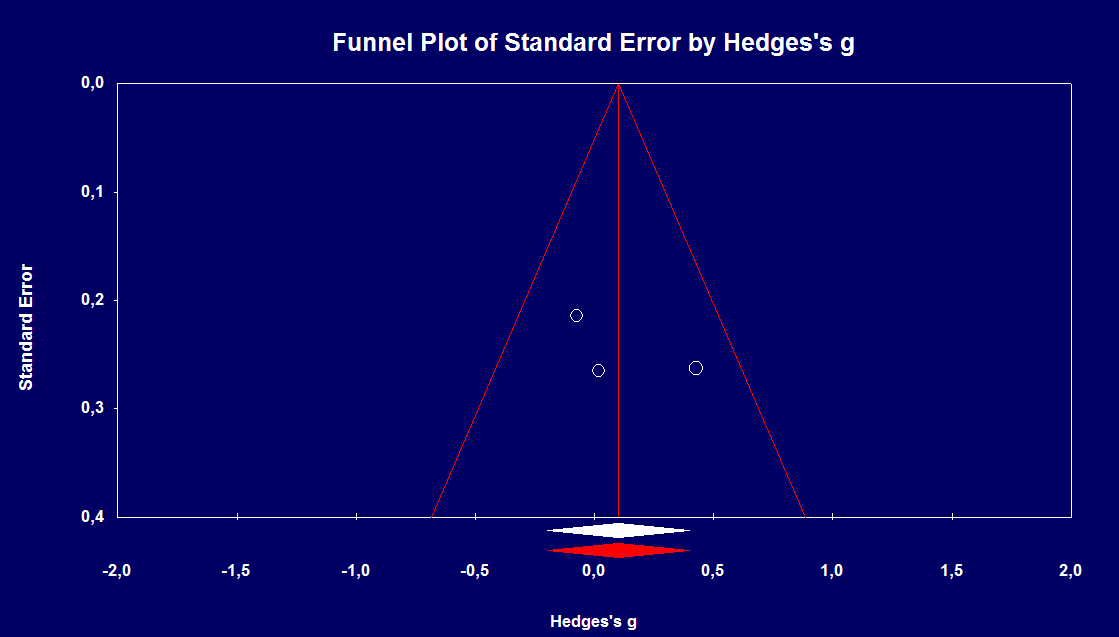

Supplement: Supplementary file 4 — Publication bias. (DOCX 111 kb) [file 12966_2019_824_MOESM4_ESM.docx]
